# A Candidate Drug for Nonalcoholic Fatty Liver Disease: A Review of Pharmacological Activities of Polygoni Multiflori Radix

**DOI:** 10.1155/2020/5462063

**Published:** 2020-04-20

**Authors:** Mengting Zhou, Naihua Hu, Meichen Liu, Ying Deng, Linfeng He, Chaocheng Guo, Xingtao Zhao, Yunxia Li

**Affiliations:** ^1^School of Pharmacy, Chengdu University of Traditional Chinese Medicine, Chengdu 611137, China; ^2^Key Laboratory of Standardization for Chinese Herbal Medicine, Ministry of Education, Chengdu 611137, China; ^3^National Key Laboratory Breeding Base of Systematic Research, Development and Utilization of Chinese Medicine Resources, Chengdu 611137, China

## Abstract

Nonalcoholic fatty liver disease, a type of metabolic syndrome, continues to rise globally. Currently, there is no approved drug for its treatment. Improving lifestyle and exercise can alleviate symptoms, but patients' compliance is poor. More and more studies have shown the potential of Polygoni Multiflori Radix (PMR) in the treatment of NAFLD and metabolic syndrome. Therefore, this paper reviews the pharmacological effects of PMR and its main chemical components (tetrahydroxystilbene glucoside, emodin, and resveratrol) on NAFLD. PMR can inhibit the production of fatty acids and promote the decomposition of triglycerides, reduce inflammation, and inhibit the occurrence of liver fibrosis. At the same time, it maintains an oxidation equilibrium status in the body, to achieve the therapeutic purpose of NAFLD and metabolic syndrome. Although more standardized studies and clinical trials are needed to confirm its efficacy, PMR may be a potential drug for the treatment of NAFLD and its complications. However, the occurrence of adverse reactions of PMR has affected its extensive clinical application. Therefore, it is necessary to further study its toxicity mechanism, enhance efficacy and control toxicity, and even reduce toxicity, which will contribute to the safe clinical use of PMR.

## 1. Introduction

Nonalcoholic fatty liver disease (NAFLD) is one of the most prevalent chronic liver diseases, especially in developed countries, and is considered to be liver manifestations of metabolic syndrome which includes obesity, hypertension, pathoglycemia, and dyslipidemia and leads to atherosclerosis, type 2 diabetes, and so on [1]. NAFLD is characterized by the abnormal accumulation of intracellular triglycerides without excess alcohol intake and is a progressive form of liver disease that includes a large range of diseases from steatosis to steatohepatitis, cirrhosis, and hepatocellular carcinoma eventually [2–4]. Histopathological examination of the occurrence of triglyceride accumulation in more than 5% of hepatocytes was defined as NAFLD [5, 6]. NAFLD is reversible in its early stage and can be intervened through lifestyle and medical treatment. If not diagnosed and treated, NAFLD may develop into nonalcoholic steatohepatitis (NASH), which may lead to irreversible liver cancer [7]. NAFLD threatens a third of the world's population, across all ages and races [8]. In China, the incidence of NAFLD continues to rise, reaching 15 percent in fast-growing cities [9]. What is worse, in recent years, research studies show that NAFLD is closely related to cognitive performance [10, 11], polycystic ovary syndrome [12], cardiovascular disease, chronic kidney disease, and other extrahepatic diseases [13]. With the change of people's unhealthy lifestyle, the incidence of NAFLD is continuously increasing, which has attracted wide attention worldwide.

At present, the main recommended treatment method is a healthy lifestyle, including strengthening physical exercise and a reasonable diet. However, according to the poor patient compliance, the treatment effect does not work well [14]. Some scholars have divided potential therapeutic drugs into four categories according to different mechanisms while they have a common goal: improving metabolic problems caused by simple fat accumulation, inhibiting nonalcoholic steatohepatitis, then alleviating liver fibrosis, and finally regulating intestinal flora to reduce intestinal fat absorption, respectively. And some drugs can have multiple effects [15]. Be that as it may, there are no approved drugs on the market for the treatment of NAFLD [16–18]. The drugs used to treat NAFLD mainly inhibit the accumulation of lipids, including insulin sensitizers and lipid-lowering drugs [19, 20]. However, insulin sensitizers have side effects such as edema and hemodilution, while statins may increase the burden on the liver [21–23]. Therefore, it is necessary to find effective therapeutic methods and drugs to control the occurrence and development of NAFLD and solve this problem.

As an important part of the world medical system, traditional Chinese medicine (TCM) has a long history in effectively various diseases. It has the characteristics of multipathway and multitarget and can be used for holistic treatment from different levels because of its remarkable curative effect and small side effects [24–26]. More and more studies show that TCM is effective in treating NAFLD [27, 28]. It is found that the extract of TCM or effective components can address not only NAFLD but also other illnesses of the metabolic syndrome, such as obesity, diabetes, and dyslipidemia. Polygoni Multiflori Radix (PMR), as a tonic medicine recorded in “Kaibao Bencao” firstly, has a history of hundreds of years in China. PMR has rich chemical compositions such as stilbenes, quinones, flavonoids, and phospholipids [29]. PMR has a wide range of pharmacological effects such as antiaging, antihyperlipidemia, anticancer, and anti-inflammatory effects, promoting immune regulation as well as nerve protection and healing, and is determined by its various components [30, 31]. Modern studies have shown that PMR has the potential to treat Alzheimer's disease, hyperlipidemia, Parkinson's disease, and inflammation. Growing evidence shows that PMR and its compounds are effective in treating NAFLD and the related complications, which is worthy of further study and discussion. Therefore, this review summarizes a series of evidence for the therapeutic action of PMR and its main components in NAFLD.

## 2. Pharmacological Effects of PMR in NAFLD

**Figure 1:**
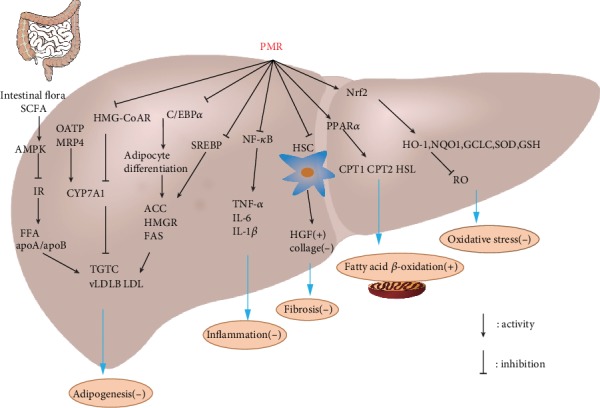
Molecular mechanism of PMR in the treatment of NAFLD. PMR exerted pharmacological effects by regulating lipid metabolism, reducing inflammation and fibrosis, improving fatty acid *β*-oxidation, alleviating oxidative stress, protecting the liver, and adjusting bile acid metabolism. PMR maintained intestinal flora homeostasis via decreasing IR and alleviating inflammation, and PMR reduced the reabsorption of fatty acids to improve NAFLD.

The diverse and complex pathogenesis of NAFLD is associated with insulin resistance (IR) which causes the excessive accumulation of free fatty acids. Without timely treatment, it may cause more serious problems such as hepatic inflammation, oxidative stress, mitochondrial dysfunction, endoplasmic reticulum (ER) stress, and apoptosis eventually which are explained by “multiple hit” hypothesis [32]. In addition to the liver injury caused by fat accumulation, the perspective of the relationship between intestinal flora and liver disease has become a research focus recently [33, 34]. Changes in the composition of intestinal microbial communities and their metabolites can also cause liver damage, such as short-chain fatty acids (SCFA), endogenous ethanol, and bile acids [35]. Therefore, maintaining intestinal flora homeostasis plays an important role in the prevention and treatment of NAFLD. The antisteatosis, antioxidation, anti-inflammation, liver protection, antiobesity, bile acid metabolism adjustment, and intestinal flora regulation effects of PMR will contribute to the treatment of NAFLD (Figure 1). More and more shreds of evidence link NAFLD to metabolic syndrome, so several aspects could be listed to state the pharmacological effects of PMR against NAFLD and metabolic syndrome according to the following seven aspects (Table 1).

### 2.1. Antisteatosis Activity

The overproduction of total cholesterol (TC) and triglyceride (TG) is considered the sign of hepatic steatosis. In the normal human body, the average content of TC and TG is 3.9 and 19.5 mg/g wet weight in the liver, respectively [6]. At the same time, hepatocytes play a vital role in biosynthesis, biodegradation of low-density lipoprotein (LDL), high-density lipoprotein (HDL), and other related lipoproteins [61, 62]. The control of hepatic steatosis is an important approach to prevent NAFLD and affect its progression to NASH, liver cirrhosis, and hepatocellular carcinoma.

PMR can regulate lipid production and metabolism to alleviate simple fatty hepatocytes. PMR and Polygoni Multiflori Radix Praeparata (PMRP) steamed with black beans showed good inhibition of hepatic steatosis. Compared to PMRP, the water extract of PMR displayed a more remarkable effect on regulating the level of TC and TG [36, 38, 40] and the effect of PMR on lipid regulation was more obvious in liver tissues of early NAFLD [37]. Research showed intuitively that PMR and PMRP could inhibit lipase with IC50 values of 38.84 *μ*g/mL and 190.6 *μ*g/mL by a bioactivity-based method, respectively [39]. PMR inhibited the formation of fat and increased the degradation of fat and the oxidation of fatty acids by upregulating the expression of peroxisome proliferator-activated receptor *α* (PPAR*α*), carnitine palmitoyltransferase 1 (CPT1), CPT2, uncoupling protein 1 (UCP1), and hormone-sensitive lipase (HSL) and downregulating adipogenic transcription factors and PPAR*γ* and diacylglycerol O-acyltransferase 2 (DGAT2) mRNA expression in 3T3-L1 preadipocyte cells and high-fat diet models [41, 42].

### 2.2. Antioxidant Activity

Oxidative stress is an imbalance of oxidation and antioxidation in the body, which produces a large number of oxide intermediates such as reactive oxygen species (ROS) and reactive nitrogen species (RNS). It leads to neutrophil inflammatory infiltration and increased protease secretion [63, 64]. Oxidative stress leads to the progression of NAFLD to NASH, exacerbating the disease [65]. Excessive fat accumulation can lead to an increase of the oxidation of fatty acids in the mitochondrion controlled by PPAR*α* and the production of excessive ROS [66]. Then, ROS mainly attacks the liver [67] and recruits Kupffer cells which can produce a variety of cytokines like tumor necrosis factor-*α* (TNF-*α*) later. As regards hepatic stellate cells, lipid peroxidation can result in proliferation and collagen synthesis caused by oxidative stress [68]. Therefore, treatment for NAFLD can be initiated by reducing oxidative stress and maintaining an antioxidant balance.

PMR has an antioxidant effect [31, 69] that protects the liver from oxidative stress and may be a potential drug for the treatment of NAFLD. PMR was often used as an antiaging drug. It was reported that the chemical profiles were applied to assess the antioxidant activities by establishing the integrated chemometric fingerprints [70]. Besides, PMR upregulated mRNA expression in the nuclear factor erythroid 2-related factor 2 (Nrf2) signal pathway including heme oxygenase-1 (HO-1), NQO1, and glutamate-cysteine ligase catalytic subunit (GCLc) dose-dependently and influenced the nuclear translocation of Nrf2 as well as reduced the content of ROS in H_2_O_2_- and acetaminophen- (APAP-) induced cells [43]. The enzyme activities of SOD, GSH, GRD, GSH-Px, and GST were improved by PMR in D-galactose-injected mice and CCl_4_-induced mice [44, 46]. PMR and PMRP improved mitochondrial *β*-oxidation by increasing the activity of CPT1A enzyme in vivo and in vitro [47].

### 2.3. Anti-inflammatory and Antifibrotic Activity

Inflammation and fibrosis can lead to the progression of simple steatosis to NASH and hepatic fibrosis. Therefore, anti-inflammation and prevention of liver fibrosis are considered a treatment direction to hold back the development of NAFLD. Inflammatory response-related signaling pathways have been reported to be the main signaling pathways for the development of liver fibrosis. Inflammation plays a major role in liver fibrosis through communication and interaction between inflammatory cells [71], cytokines [72, 73], and related signaling pathways [74].

PMR could regulate inflammatory mediators and inflammatory transcription factors like nuclear factor kappa-B (NF-*κ*B) for anti-inflammatory purposes. The results proved that the ethanol extract of PMR had an anti-inflammatory effect. The extraction of PMR reduced the expression of TNF-*α*, GST-*α*, and interleukin 6 (IL-6) which were regarded as therapeutic targets for hepatic inflammation or fibrosis in high-fat diet (HFD) rats [41, 42]. In CCl_4_-induced in vivo and in vitro models, PMR remarkably decreased the content of TNF-*α* [48]. NF-*κ*B was an important immune-related transcription factor that regulated many cytokines and adhesion factors. PMR inhibited the NF-*κ*B transcriptional activity in TNF-*α*-induced NF-*κ*B activation compared with the model group evaluated by luciferase reporter gene assays [49]. PMR significantly inhibited the activation of hepatic stellate cells induced by PDGF and facilitated the phagocytic activity of Kupffer cells in a concentration-dependent manner [50]. In CCl_4_-induced rats of liver fibrosis, the water extract of PMR improved serum albumin which was an indicator of chronic liver damage and reduced the pathological grade of liver fibrosis as well as the occurrence of ascites [51].

### 2.4. Hepatoprotective Activity

Patients with NAFLD show elevated levels of ALT and AST, which are important biochemical indicators of liver injury. Without timely treatment and control, NAFLD can progress into cirrhosis.

PMR could alleviate the damage to the liver and might become a hepatoprotective medicine to treat NAFLD. The extract of PMR reduced the contents of AST and ALT in serum [42–44, 48] and the production of malondialdehyde (MDA) compared with CCl_4_-induced liver damage. In addition, the TNF-*α* was reduced and histopathology examination showed relieved adipose tissue and necrosis in the PMR treatment group [48]. It not only increased the hepatocyte growth factor (HGF) which played an important role in liver regeneration and attenuated development of liver cirrhosis but also increased hydroxyproline that was an indicator for collagen content. Consequently, the survival rate was enhanced largely in the PMR treatment group [50].

### 2.5. Hypolipidemic Activity

Hyperlipidemia is a common metabolic syndrome associated with increased TC, TG, and LDL-C, while decreased HDL-C levels [75]. An overload of cholesterol in the liver can lead to fatty liver disease. Therefore, regulating cholesterol balance is an effective means to treat NAFLD.

PMR might control the development of NAFLD by regulating abnormal markers of cholesterol which indicated the severity and progression of NAFLD. Traditional Chinese medicine prescriptions containing PMR have been used for many years to treat NAFLD and hyperlipidemia such as Xuezhining Wan and Shouwu Wan [76]. The extraction of PMR showed a remarkable increase in the activities of 3-hydroxy-3-methylglutaryl-CoA reductase (HMGR) related to TC biosynthesis; meanwhile, fatty acid synthase (FAS) and acetyl-CoA carboxylase (ACC) decreased sufficiently which played an important role in the biosynthesis of TG [41]. However, researchers found that PMRP was more effective in regulating lipids in circulating blood to treat hyperlipidemia [37]. In addition, PMR lowered the plasma LDL-C, TC, and TG levels in high-fat diet rats [41, 55] and hyperlipidemia patients [52–54, 56].

### 2.6. Antiobesity Activity

Due to people's unhealthy lifestyle, obesity is prevalent all over the world. It is accompanied with many health problems including dyslipidemia, type 2 diabetes, and steatosis [77]. Obesity leads to various metabolic abnormalities, and the proliferation of adipose tissue is closely related to the imbalance of various transcription factors [78].

Based on the antiobesity effect of PMR, it might be developed as a potential weight loss drug to replace the existing weight loss agents with large side effects. In order to reduce the accumulation of fat in the body, lipase inhibitors have been selected as targets to prevent the digestion and absorption of fat for the treatment of obesity [79]. Studies have shown that many ingredients in PMR were screened for potential lipase inhibitors such as stilbenes and anthraquinones, which could be used for curing obesity [39]. 70% ethanol extract of PMR could not only reduce weight but also reduce visceral fat weight including epididymal, retroperitoneal, perirenal, and mesenteric white adipose tissue in HFD-induced obese mice. PMR reduced the expression of CCAAT/enhancer-binding protein *α* (C/EBP*α*) and PPAR*γ* which played vital roles in controlling the number and size of fat cells. Meanwhile, the expression of FAS also decreased in 3T3-L1 preadipocyte cells cured by PMR [42, 57].

### 2.7. Intestinal Flora Regulatory Activity

Intestinal flora is closely related to the development of NAFLD [80]. The accumulating evidence suggests that changes in intestinal flora can promote the deterioration of NAFLD by influencing processes of inflammation, bile acids, and IR, and vice versa [81, 82]. And intestinal flora promotes the development of NAFLD through the enterohepatic axis [83]. SCFA are metabolites produced by intestinal flora rather than the host [84] mainly including acetic acid, propionic acid, and butyric acid, which can mediate the inflammatory response through various channels and directly or indirectly affect NAFLD.

PMR can regulate NAFLD by maintaining intestinal flora homeostasis to change bile acid metabolism and fatty acid absorption. The extraction of PMR could decrease the content of TC and TG in the liver tissue of NAFLD mice fed with a high-fat diet; at the same time, it reduced the total SCFA in the intestinal canal of the model group. However, there were gender differences in the change of different SCFA [58]. PMR could regulate blood glucose and alleviate IR by managing the diversity of intestinal flora such as changing the imbalance of Firmicutes/Bacteroides which was directly proportional to the level of blood sugar [59, 85] and the relative abundance of Proteobacteria and so on [60].

## 3. Pharmacological Effects of Active Constituents of PMR in NAFLD

**Figure 2:**
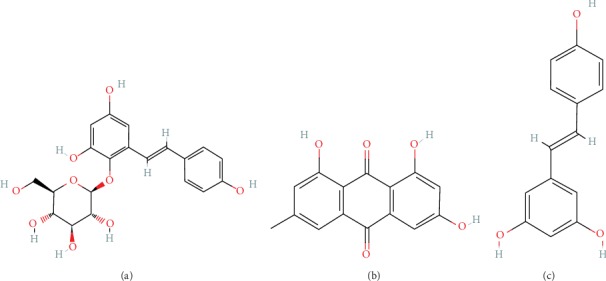
Chemical structures of three constituents from PMR. (a) tetrahydroxystilbene glucoside, (b) emodin, and (c) resveratrol.

There are 133 chemical constituents isolated from PMR, including stilbene glycosides, anthraquinones, flavonoids, phospholipids, and phenylpropanoids [86]. Stilbene glycosides and anthraquinones are the main components of PMR. Studies have shown that tetrahydroxystilbene glucoside, emodin, and resveratrol can effectively improve NAFLD (Figure 2). Therefore, this paper reviews the therapeutic effects of the three components in NAFLD (Table 2).

### 3.1. Tetrahydroxystilbene Glucoside

Tetrahydroxystilbene glucoside, named 2,3,5,4′-tetrahydroxystilbene-2-O-*β*-D-glucoside (TSG), is the main component extracted from PMR. It is regarded as a quality control indicator of PMR and is required to contain no less than 1% in Chinese Pharmacopoeia. There was growing evidence that TSG had a wide range of pharmacological effects such as anti-inflammation, antioxidation, and antiapoptosis [124, 125].

TSG attenuated the inflammatory response by downregulating the levels of IL-6 and TNF-*α* in HFD-induced apoE^−/−^ mice. In vivo experiment showed that TSG significantly reduced the release of inflammatory factors IL-6, TNF-*α*, and C-reactive protein in high-fat and high-cholesterol diet rats [89, 90]. Besides, TSG decreased the expression of p-Smad3 that increased NF-*κ*B inhibitor I*κ*B*α* degradation and then promoted the activation of the NF-*κ*B signaling pathway. 3-Hydroxy-3-methylglutaryl coenzyme A (HMG-CoA) reductase inhibitors could increase LDL-C uptake and metabolism by increasing the number of LDL receptors on the surface of hepatocytes [126]. TSG could reduce the LDL level by increasing the expression of LDL receptors and TC and TG in hyperlipidemic rats and increase the HDL [45, 89–93]. Hence, TSG may be used as HMG-CoA reductase inhibitors to decrease the level of LDL. In fat emulsion-incubated L-02 cells, TSG effectively reduced the accumulation of triglycerides by inhibiting the expression of related proteins that synthesized triglycerides [87, 88]. Reverse cholesterol transport (RCT) was involved in cholesterol metabolism by transporting cholesterol to the liver; TSG mediated the RCT signaling pathway by upregulating the expression of ATP-binding cassette transporter A1 (ABCA1), ABCG1, and scavenger receptor class B type I (SR-BI) which regulated cholesterol efflux from the macrophage [127] and the expression of cholesterol 7*α*-hydroxylase (CYP7A1) that was a rate-limiting enzyme of bile acid synthesis [128]. Therefore, the lipid profiles decreased owing to the increased level of excretion [92]. In a study after HFD rats were orally administrated with TSG, the activity of SOD, CAT, GSH-Px, and T-AOC was increased remarkably indicating that TSG had an antioxidant effect to cure hyperlipidemia [90]. Particularly, TSG downregulated the expression of *α*-SMA associated with the activation of hepatic stellate cells, and TNF*β* correlated to the fibrosis-related genes [45, 90]. Studies have shown that TSG could also regulate the homeostasis of intestinal flora to rectify lipid metabolism by increasing Akkermansia genera and the ratio of Firmicutes/Bacteroidetes, while the abundance of Helicobacter pylori decreased [59].

**Figure 3:**
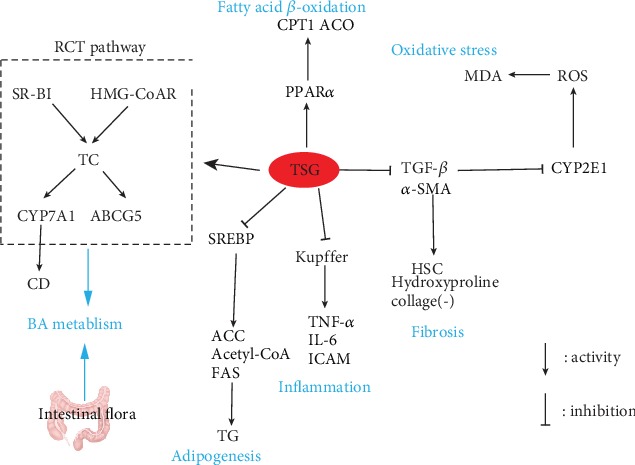
Schematic illustration of mechanism of TSG on improving NAFLD. TSG could not only improve bile acid metabolism abnormalities caused by NAFLD through the PCT signaling pathway and intestinal flora but also inhibit fat production, inflammation, and oxidative stress pathways, while promoting the *β*-oxidation of fatty acids.

Taken together, TSG might develop as an underlying agent against NAFLD through mediating liver lipid metabolism, alleviating inflammation, regulating oxidation and fibrosis, and other ways (Figure 3).

### 3.2. Emodin

Emodin (1,3,8-trihydroxy-6-methylanthraquinone) is a hydroxyanthraquinone derivative in PMR and has a wide range of physiological activities. The experimental results demonstrated that it has anti-inflammatory, antioxidant, hepatoprotective, and anticancer activities [129–131]. There was growing evidence that emodin had a significant effect on the treatment of NAFLD.

Emodin alleviated the lipid accumulation and ameliorated hepatic steatosis in vivo and in vitro [96, 97]. It reduced the expression of sterol regulatory element-binding protein 1 (SREBP1) [95] which was an important lipogenic transcription factor associated with triglyceride accumulation [132] and the phosphorylated mTOR (p-mTOR) that positively regulated the activity of SREBP1, while the expression of AMP-activated protein kinase (AMPK) which was an indirect upstream kinase of SREBP1 was increased. Hence, emodin effectively regulated lipid metabolism via the CaMKK-AMPK-mTOR-p70S6K signaling pathway [94]. Furthermore, emodin inhibited the expression of HMG-CoA reductase and DGAT1 associated with the synthesis of TC and TG [88]. In addition, emodin showed a powerful effect on lowering blood lipids by inhibiting the activity of SMase, the content of CRE, and the quantity of apoptotic foam cell and promoting antioxidant ability at the same time [96, 98]. Emodin also alleviated inflammation by reducing leukocyte infiltration as well as the expression of inflammatory factors. Further study showed that extracellular regulated protein kinases 1/2 (Erk1/2), p38, toll-like receptor 4 (TLR4), and NF-*κ*B signaling pathways were inhibited dramatically [96, 100, 101], so we could conclude that emodin could make a contribution to steatohepatitis in a way. However, it was reported that emodin could aggravate liver damage and inflammation in MCD diet-induced NAFLD in mice along with the increased serum ALT and AST levels and the expression of inflammatory factors IL-1*β* and IL-6 [99]. Two completely opposite results might be due to the different modeling methods and model animals, which needed further exploration. In addition, emodin improved liver fibrosis via decreasing transforming growth factor-*β*1 (TGF-*β*1) to inhibit the activation of hepatic stellate cells and the infiltration of Gr1^hi^ monocytes [102–104].

**Figure 4:**
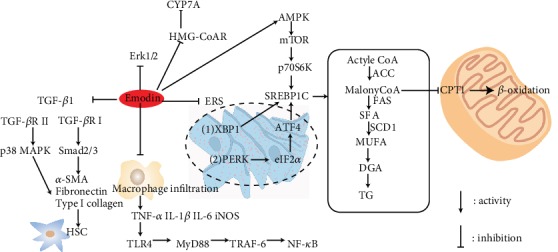
Schematic illustration of mechanism of emodin on improving NAFLD. Emodin mainly reduced fat production and increased *β*-oxidation of fatty acids by inhibiting the oxidative stress of the endoplasmic reticulum and alleviated the inflammatory response by inhibiting the Erk1/2, p38, and NF-*κ*B signaling pathway. Emodin suppressed the activation of hepatic stellate cells via inhibiting the expression of TGF-*β*1.

In conclusion, the emodin could develop into a potential agent to prevent the progression of NAFLD to NASH owing to a variety of pharmacological activities (Figure 4).

### 3.3. Resveratrol

Resveratrol is a polyphenol named trans-3,5,4′-trihydroxy-trans-stilbene (RES). Resveratrol had both *cis* and *trans* optical isomers, and studies had shown that the latter was more stable and active [133]. A large number of studies had shown that it had a powerful effect on the prevention and treatment of NAFLD.

Most of the available experimental data came from two models including in vivo experiments of mice or rats with high-fat diet as well as in vitro tests with primary hepatocytes or HepG2 cells. RES could improve the symptom of NAFLD by protecting the liver, adjusting lipid metabolism, alleviating inflammation and fibrosis, regulating the oxidation equilibrium status, and enhancing autophagy [119, 120, 122] as well as controlling the farnesoid X receptor (FXR) [123]. The increase of serum TC, TG, LDL-C, ALT, and AST content and the reduction of HDL-C were as serum markers of NAFLD, and RES could return them to normality effectively due to the hepatoprotective and lipid metabolic activity [105–107, 111, 113]. In addition, in FFA-, PA-, OA-, or HG-induced HepG2 cell models, RES reduced lipid droplet accumulation indirectly [108, 110, 112]. Sirtuin 1 (SIRT1) was an important regulator associated with glucose and fat acid metabolism in the liver. A study showed that RES could remarkably activate the expression of SIRT1. At the same time, a series of proteins related to lipid droplets were downregulated such as activating transcription factor 6 (ATF6), cAMP response element-binding protein H (CREBH), and perilipin 1 (PLIN1) [109, 116]. Liver inflammation was accompanied with the increase of inflammatory cytokines. RES reduced the expression and secretion of proinflammatory cytokines (IL-1*β*, IL-6, TNF-*α*, and TLR4), and further studies also suggested that RES suppressed NF-*κ*B which was a transcription factor combined with its inhibitor I*κ*B*α* and bound to DNA and then promoted the expression of cytokines when it was activated by an external stimulus [134] via activating the phosphorylation of AMPK*α* and the expression of SIRT1 [116–119]. In addition, RES reduced collagen fiber bundles, hydroxyproline, and lysyl oxidase (LOX) to alleviate liver fibrosis [121]. A lot of evidence showed RES improved redox balance by activating PPAR*α* related to fatty acid oxidation and inhibiting SREBP1c associated to lipogenesis [106, 110, 114]. Meanwhile, the content and activity of T-SOD and GPx were improved by the treatment with RES, while the content of MDA decreased [107]. The Nrf2-Keap1 pathway participated in the prevention of metabolic disorders in NAFLD, and RES could activate Nrf2 signaling to inhibit lipogenesis [115]. However, RES presented low bioavailability due to poor solubility. Many researchers were devoted to exploiting new dosage forms, for example, the PLGA nanoparticles loaded with RES, in order to improve the effect [135, 136].

**Figure 5:**
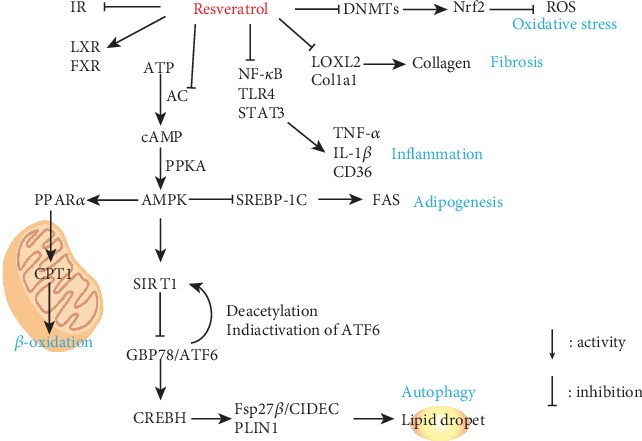
Schematic illustration of mechanism of RES on improving NAFLD. RES promoted autophagy to reduce the accumulation of lipid droplets and inhibited *β*-oxidation of fatty acids by activating the AMPK signaling pathway. RES alleviated the symptom caused by inflammation, liver fibrosis, and oxidative stress and improved the expression of LXR and FXR genes.

To sum up, RES had varieties of biological activities, which have been proved to play a potential role in treating NAFLD (Figure 5).

## 4. Knowledge of Toxicity

With the widespread application of PMR and its preparations, adverse reactions related to the hepatotoxicity of PMR have been reported in the early 20th century [137–139]. Therefore, the toxicity of PMR attracted wide attention. The adverse reactions of PMR were jaundice, yellowing urine, cholestasis, liver injury, etc. It was reported that the toxicity of the ethanol extract of PMR was higher than that of the water extract. Therefore, it was not recommended to make wine with PMR for nourishing. 70% ethanol extract had the highest toxicity [140]. Studies showed that the occurrence of adverse reactions was related to time and dose, and a long-term large dose was more likely to cause hepatotoxicity [139]. The other researchers thought that PMR-induced hepatic injury was an idiosyncratic drug-induced liver injury, so they built the lipopolysaccharide- (LPS-) induced model of hepatotoxicity [141–143] to explore the toxic substance basis and mechanism of PMR.

Many claims have been made to clarify its toxic components. Some studies concluded that adverse reactions were mainly due to its anthraquinone components [31, 144, 145]. Emodin and its derivatives were the most likely hepatotoxic components [144] and had a time-dependent intracellular accumulation [146], while TSG and physcion may mitigate the effects of emodin [147]. Nevertheless, some studies held that the hepatotoxicity of PMR depended not only on the composition of emodin but also on the content of TSG [147]. Therefore, there are many uncertainties about the toxic components of PMR, and more toxicological studies are needed.

The clinical application of PMR pays attention to compatibility, and reasonable compatibility can reduce toxicity. PMR can be used with other TCM to increase the curative effect and reduce toxicity. At the same time, its toxicity may be attributed to high doses and prolonged use. Clinical use of PMR should attach great importance to the examination of liver function. PMRP toxicity is more suitable for safe clinical use with lower toxicity [147]. In the meantime, it is important to improve the public's correct understanding of PMR.

## 5. Discussion

PMR, based on the theory of TCM, PMR, and PMRP, has different effects. PMR can moisten intestines and help defecate, remove toxicity, and eliminate carbuncles, while PMRP which is steamed with black soya beans has a large effect including nourishing the liver and kidney, strengthening bones and muscles, and blackening the beard and hair. Modern studies have shown that PMR and PMRP had therapeutic potential for aging, hair loss, hyperlipidemia, inflammation, and cancer [148]. Numerous experimental data indicated the potential of PMR in the treatment of NAFLD. In the experiments, scholars found that both PMR and PMRP could effectively reduce TC, TG, and LDL-C and increase HDL-C content to regulate lipid metabolism. The effective compounds, TSG, emodin, and resveratrol, might have synergic effects in the body to regulate lipid metabolism in NAFLD. However, studies have found that PMR had a better effect on lowering lipids [38, 39, 76]. The possible reason is that after processing, the content and proportion of active ingredients are changed. For example, conjugated anthraquinone compounds are hydrolyzed at high temperature to increase the content of free anthraquinone [149]. At the same time, the content of TSG decreased significantly [29]. Treatment of the NAFLD process is complicated by PMR because of its rich pharmacological effects and complex ingredients. It can go through the different ways in the different mechanisms to realize regulation by the multicomponent and multiple targets.

To sum up, the therapeutic mechanism of PMR is mainly controlled by the following pathways: (1) reducing lipid formation by downregulating SREBP, ACC, and FAS; (2) suppressing the release of inflammatory cytokines through the NF-*κ*B signaling pathway; (3) resulting in antifibrosis by inhibiting the activation of hepatic stellate cells; (4) augmenting fatty acid *β*-oxidation via upregulating the PPAR*α*; (5) reducing oxidative stress and improving antioxidant levels through Nrf2; (6) reducing IR and improving bile acid metabolism by regulating intestinal flora and increasing the expression of CYP7A1; and (7) decreasing ALT and AST levels to protect the liver. These different pathways work together to improve NAFLD by regulating lipid metabolism, reducing inflammation and fibrosis, improving antioxidant levels, and protecting the liver.

This review also summarizes the research progress of the three main components of PMR in the treatment of NAFLD. TSG, emodin, and RES whose pharmacological activities are consistent with those of PMR all show antisteatosis, anti-inflammatory, antifibrotic, and antioxidative stress activities and increase *β*-oxidation of fatty acids in mitochondria. Meanwhile, TSG and emodin can regulate bile acid metabolism by increasing the expression of CYP7A1, while RES can affect bile acid metabolism by regulating LXR and FXR genes which can adjust CYP7A1 indirectly [150]. Therefore, these three components may contribute to the activity of PMR in regulating bile acid metabolism. Of these three components, the current literature has found that only RES has been shown to reduce lipid droplet accumulation by upregulating SIRT1 to activate the autophagy pathway. However, studies on PMR have not mentioned the reduction of lipid droplets through autophagy. The possible reason is that the content of RES in PMR is low, and the administration of the PMR extract does not reach the concentration to render autophagy, while the administration of the RES monomer has obvious effects.

## 6. Conclusion

This review describes in detail the therapeutic effects of PMR and its chemical components on NAFLD. Its antisteatosis, antioxidation, anti-inflammation, antifibrosis, liver protection, lipid reduction, antiobesity, intestinal flora regulation, and bile acid adjustment effects might contribute to its therapeutic effects. Although stilbene glycosides and anthraquinones are the main components, the relationship between the two is still unclear; whether they act synergically or inhibit each other and the sequence of action need further study. At present, adverse reactions of PMR are frequent, but its therapeutic effect is undeniable. Therefore, it is necessary not only to understand the basis and mechanism of its efficacy in the treatment of NAFLD but also to further study its toxicity mechanism so as to contribute to the safety and wide use of PMR in clinical practice.

## Figures and Tables

**Table 1:** Pharmacological activities of Polygoni Multiflori Radix in NAFLD.

Pharmacological effects	Extraction solvent	Country	Model	Efficient doses	Results	References
Antisteatosis activity	Water	China	1% fat emulsion induced L-02 cells	10, 20, 40, 80, 100 *μ*g/mL	TG↓, TC↓	[36]
	Water	China	High-fat diet rats	0.405, 0.810, 1.62 g/kg	Liver TG↓, TC↓, LDL-C↓	[37]
	50% ethanol	China	High-fat diet rats	10.5, 3.5, 1.17 g/kg	TC↓, TG↓, LDL-C↓, HDL-C↑	[38]
	Ethanol	China		4-5000 *μ*g/mL	Inhibit lipase	[39]
	Water	China	CCl_4_, cortisone, acetate, TAA-induced mice	15 g/kg	The enlargement of liver↓, TG↓	[40]
	70% ethanol	China	High-fat diet rats	2.7, 8.1, 16.2 g/kg	HMGR, FAS, ACC, SREBP1↓, TC, TG, LDL-C↓	[41]
	70% ethanol	Korea	High-fat diet mice, TCA-treated3T3-L1 preadipocyte cells	0.05%; 10, 30, 50, 100 *μ*g/mL	C/EBP*α*, PPAR*γ*, FAS, body weight, DGAT2↓, PPAR*α*, CPT1, CPT2, UCP1, HSL↑	[42]

Antioxidant activity	50% ethanol	China	APAP, H_2_O_2_-treated HepG2	20, 50, 100 *μ*g/mL	HO-1, NQO1, GCLc mRNA↑, Nrf2 in nuclear fraction↑, Nrf2 in cellular fraction↓, ROS↓, superoxide anion↓, MRP4↑, survival rate↑, OATP↓	[43]
	75% ethanol	China	D-Galactose-injected mice	1, 0.6, 0.3 mL/kg	SOD↑, GSH-Px↑	[44]
	70% ethanol	China	High-fat diet rats	12, 24 mg/kg	MDA↓, SOD, CAT, GSH-Px, T-POC↑	[45]
	Ethyl acetate	China	CCl_4_-induced mice	0.5-1.5 g/kg	GSH, GRD, GSH-Px, GST↑, plasma ALT, SDH, MDA↓	[46]
	Water	China	High-fat diet rats; NEFA-inducedL-02 cells	70, 140, 280 mg/kg; 3.75, 7.5, 15, 30, 60 *μ*g/mL	ALT, AST, ROS, TC, TG, lipid droplets↓, mitochondrial *β*-oxidation, CPT1A↑	[47]

Anti-inflammatory and antifibrotic activity	70% ethanol	China	High-fat diet rats	2.7, 8.1, 16.2 g/kg	TNF-*α*, GST-*α*↓	[41]
	70% ethanol	Korea	High-fat diet mice	0.05%	IL-6, TNF-*α*↓, leptin, ALT, AST↓	[42]
	Water	China	CCl_4_-induced rat; CCl_4_-induced BCRC 60201 cells	200, 400 mg/kg; 50–300 *μ*g/ mL	TNF-*α*↓, fatty degeneration, and necrosis↓	[48]
	70% ethanol	Korea	TNF-*α*-induced HepG2 cells	0.1, 1, 10 *μ*M	NF-*κ*B transcriptional activity↓	[49]
	Methanol	China	DMN-induced mice, hepatic nonparenchymal cells	1–1000 g/mL	HGF, the phagocytic activity of liver Kupffer cells, survival rate↑, proliferation of hepatic stellate cells, hydroxyproline↓	[50]
	Water	China	CCl_4_-induced rats	10 mL/kg	ALB↑, the ratio of ascites, the degree of fibrosis↓	[51]

Hepatoprotective activity	70% ethanol	Korea	High-fat diet mice	0.05%	AST↓	[42]
	50% ethanol	China	APAP-induced mouse	120 mg/kg	Plasma AST, ALT↓	[43]
	75% ethanol	China	D-Galactose-injected mice	1, 0.6, 0.3 g/mL/kg	ALT↓, AST↓, MDA↓	[44]
	Water	China	CCl_4_-induced rat; CCl_4_-induced BCRC 60201 cells	200, 400 mg/kg; 50–300 *μ*g/mL	ALT↓, AST↓, MDA↓, glutathione S-transferase and catalase activity↑, serum ALT, AST, MDA↓	[48]
	Methanol	China	Dimethylnitrosamine-induced mice	20, 100 mg/kg	Hydroxyproline↓, hepatocyte growth factor (HGF)↑, survival rate↑	[50]

Hypolipidemic activity	Water	China	High-fat diet rats	0.810, 1.62, 3.24 g/kg	TC, HDL-C↓	[37]
	70% ethanol	China	High-fat diet rats	2.7, 8.1, 16.2 g/kg	Plasma LDL-C, TC, TG, HMGR, FAS, ACC↓	[41]
	70% ethanol	China	High-fat diet rats	12, 24 mg/kg	TC, TG, LDL-C↓, apoA/apoB, HDL-C/TC↑	[45]
	Water	China	Hyperlipidemia patients	10 g/d	TC, TG↓, apoA/apoB↑	[52]
	Water	China	Hyperlipidemia patients	150 mL × 2/d	TC, TG, LDL↓, HDL↑	[53]
	Water	China	Hyperlipidemia patients	3 g/d	TC, TG, LDL↓, HDL↑	[54]
	Water	China	High-fat diet rats	PMR 0.4050, 0.8100, 0.1620; PMRP 0.8100, 0.1620, 3.240 g/kg	TC, TG, VLDL, the activity of DGAT↓, HL↑	[55]
	Water	China	High-fat diet rats	5 mL/d	TC, TG↓	[56]

Antiobesity activity	70% ethanol	Korea	3T3-L1 cells; high-fat diet mice	5, 10 *μ*g/mL; 0.05%	3T3-L1 differentiation, lipid accumulation, TG, C/EBP*α*, PPAR*γ*, FAS↓; body weight, leptin↓	[42]
	40% ethanol	China	Rats	2 mL	The activity of FAS, body weight↓	[57]

Intestinal flora regulatory activity	Water	China	High-fat diet rats	405, 810 mg/kg	TC, TG, LPS, total SCFA, acetic acid, propionic acid, butyric acid↓	[58]
	Water	China	High-fat diet mice	1.125 mg/g	Firmicutes/Bacteroidetes↑	[59]
	80% ethanol	China	High-fat and sugar diet rats	57, 228 mg/kg; 12, 48 mg/kg	Firmicutes/Bacteroides↑, Clostridium spp., Desulfovibrio spp.; Oscillibacter spp.↓, Bacteroides spp., Bifidobacterium spp.↑	[60]

**Table 2:** Pharmacological activities of tetrahydroxystilbene glucoside, emodin, and resveratrol in NAFLD.

Pharmacological effects	Country	Type	Doses	Model	Results	References
Tetrahydroxystilbene glucoside						
Antisteatosis activity	China	In vitro	150 *μ*M/L	Fat emulsion-induced L-02 cells	TG, SREBP1c, ACACA, FASN, FATP4, L-FABP↓, PPAR*α*↑	[87]
	China	In vitro	50-300 *μ*M	Fat emulsion-induced L-02 cells	TC, TG, HMG-CoA reductase↓, CYP7A↑	[88]
Anti-inflammatory activity	China	In vivo	0.035, 0.07 mg/g	HFD-induced mice	IL-6, TNF-*α*, VCAM-1, MCP-1, TG, ox-LDL↓	[59]
	China	In vivo	30, 60, 120 mg/kg	HFD/HCD-induced rats	IL-6, TNF-*α*, CRP↓	[89]
	China	In vivo	50, 100 mg/kg	HFD-induced mice	CD68, TNF-*α*, IL-6, ICAM↓	[90]
Hypolipidemic activity	China	In vivo	12, 24 mg/kg	HFD-induced rats	TC, TG, LDL-C, apoB, MDA↓	[45]
	China	In vivo	120, 60, 30 mg/kg	HFD/HCD-induced rats	TC, TG, LDL↓, HDL↑	[89]
	China	In vivo	50, 100 mg/kg	HFD-induced mice	TC, TG, LDL-C↓, HDL-C↑; ALT, AST↓, SREBP1c, ACC*α*, FAS↓, PPAR*α*, CPT1A, ACO, ABCG5, CYP7A1↑	[90]
	China	In vivo	90, 180 mg/kg	Hyperlipidemic rats	TC, LDL-C, AI↓, LDLR↑	[91]
	China	In vivo	50, 100 mg/kg	HFD-induced apoE^−/−^ mice	TC, TG, LDL, ↓ABCA1, ABCG1, HDL, SR-BI, ABCG5, CYP7A1↑	[92]
	China	In vivo	30, 60, 120 mg/kg	HFD-induced rats	TC, TG, LDL-C, MDA, TC/HDL-C↓	[93]
Antioxidant activity	China	In vivo	12, 24 mg/kg	HFD-induced rats	SOD, CAT, GSH-Px, T-AOC↑	[45]
	China	In vivo	50, 100 mg/kg	HFD-induced mice	ROS, NOX-2, NOX-4, CYP2E1, MDA↓, SOD, GSH, CAT↑	[90]
Antifibrotic activity	China	In vivo	50, 100 mg/kg	HFD-induced mice	*α*-SMA and TGF-*β*↓	[90]
Intestinal flora regulatory activity	China	In vivo	0.035, 0.07 mg/g	HFD-induced mice	Bacteroidetes, Proteobacteria, Tenericutes, Helicobacter pylori↓, Firmicutes, Akkermansia↑	[59]

Emodin						
Antisteatosis activity	China	In vitro	50-300 *μ*M	Fat emulsion-induced L-02 cells	HMG-CoA reductase, DGAT1↓, CYP7A↑	[88]
	China	In vivo and in vitro	20, 40, 80 *μ*M; 40, 80, 160 mg/kg	FFA-induced HepG2 cells; HFD-induced rats	Intracellular lipids, TC, TG, SREBP1, SCD1, FAS, CD36, p-mTOR, P-p70S6K↓, CPT1, PPAR*α*, P-AMPK, P-ACC↑	[94]
	China	In vivo	40, 80, 160 mg/kg	Fructose-induced rats	SREBP1c, body weight, liver index, serum and hepatic TG, ACC1, FAS, SCD1, GRP78↓, CPT1, SREBP1c↑	[95]
	Italy	In vivo	40 mg/kg	HFD/HF-induced rats	TG, ALT, glucose, insulin, HOMA-IR↓	[96]
	China	In vivo	40 mg/kg	HCD-induced rats	Body weight, liver index, serum ALT, blood lipids, hepatic triglyceride↓, PPAR*γ*↑	[97]
Antioxidant activity	Italy	In vivo	40 mg/kg	HFD/HF-induced rats	Pro SSG/Tot GSH, PTEN phosphorylation/glutathionylation	[96]
	China	In vivo	10 mg/kg	HFD/HF-induced rats	SMase, CRE, apoptotic foam cell, MDA, OxLDL↓, SOD↑	[98]
Anti-inflammatory activity	Italy	In vivo	40 mg/kg	HFD/HF-induced rats	TNF-*α*↓	[96]
	China	In vivo	40 mg/kg	MCD-induced mice	ALT, AST, IL-1*β*, IL-6↑	[99]
	USA.	In vivo and in vitro	40 mg/kg; 25 *μ*M	LPS-induced hyperlipidemic mice and macrophages	Liver weight, total liver infiltrating cells, liver infiltrating cells, leukocyte number, ALT, AST, ORO positive area, cholesterol↓; TNF-*α*, IL-6, IL-1*β*, iNOS, P-Erk/t-Erk↓	[100]
	China	In vivo	20, 40, 80 mg/kg	HFD-induced rats	TNF-*α*, IL-1, TLR4, MyD88, TRAF-6↓	[101]
Antifibrotic activity	China	In vitro	3, 10, 30 *μ*M	SB203580 and TGF-*β*-neutralizing antibody-treated HSC-T6 cells	*α*-SMA, fibronectin, type I collagen, TGF-*β*1, TGF-*β*R I, TGF-*β*R II↓	[102]
	China	In vivo	10, 20, 40 mg/kg	CCl_4_-induced rats	Collagen, TGF-*β*1↓	[103]
	China	In vivo	20 mg/kg	CCl_4_-induced mice	TGF-*β*1, IL-1*β*, TNF-*α*, GRN, MCP-1, CCL7↓	[104]

Resveratrol						
Antisteatosis activity	China	In vivo	15 mg/kg	HFD-induced rats	TC, TG, LDL-C↓, HDL-C↑	[105]
	China	In vivo and in vitro	100 mg/kg; 40 *μ*M	HFD-induced rats; PA-induced HepG2 cells	Body weight, liver index, TC, TG, LDL-C↓	[106]
	China	In vivo	100 mg/kg	HFD-induced mice	TC, HDL-C, glucose, insulin, HOMA-IR↓	[107]
	Poland	In vitro	10, 20 *μ*M	HG-induced HepG2 cells	Lipid accumulation↓	[108]
	China	In vivo and in vitro	400 mg/kg; 10, 20, 40 *μ*M	HFD-induced mice; PA-induced HepG2 cells	SIRT1↑, ATF6, Fsp27*β*/CIDEC, CREBH, PLIN1↓	[109]
	Poland	In vitro	10, 20 mol/L	OA- and PA-induced HepG2 cells	Lipid accumulation↓	[110]
	Serbia	In vivo	20 mg/kg	Cholesterol and cholic acid-induced rats	HDL↑, LDL, TG↓	[111]
	China	In vivo and in vitro	15 mg/kg; 20 *μ*M	HFD-induced mice; FFA-induced HepG2 cells	TG, body weight, lipid accumulation, ROS↓	[112]
Hepatoprotective activity	China	In vivo	15 mg/kg	HFD-induced rats	ALT, AST, TBIL, DBIL, IBIL↓	[105]
	China	In vivo and in vitro	100 mg/kg; 40 *μ*M	HFD-induced rats; PA-induced HepG2 cells	ALT, AST↓	[106]
	China	In vivo	100 mg/kg	HFD-induced mice	ALT, AST↓	[107]
	Cyprus	In vivo	50 mg	Patients	SGPT, g-GT, IR↓	[113]
Antioxidant activity	China	In vivo and in vitro	100 mg/kg; 40 *μ*M	HFD-induced rats; PA-induced HepG2 cells	SREBP1c, FAS, mtROS↓, PPAR*α*, AMPK↑	[106]
	China	In vivo	100 mg/kg	HFD-induced mice	MDA, T-SOD, GPx, CD36↓	[107]
	Poland	In vitro	10, 20 mol/L	OA- and PA-induced HepG2 cells	Apoptotic cells, oxidative stress intensity↓, mitochondrial membrane potential↑	[110]
	Egypt	In vivo	20 mg/kg	HFD-induced rats	Proteolytic cleavage of SREBP1 and SREBP2, CPT1, UCP2↓	[114]
	Iran	In vivo and in vitro	0.4%; 20 *μ*M	HFD-induced mice; HD-induced HepG2 cells	Nrf2, HO-1, NQO1, SOD↑, TG, FAS, FBS, SREBP1c↓	[115]
Anti-inflammatory activity	China	In vivo	100 mg/kg	HFD-induced mice	TNF-*α*, TLR4↓	[107]
	China	In vivo and vitro	30 mg/kg; 50, 100 *μ*M	HFD-induced mice; NEFA-induced primary hepatocytes of mice	IL-1*β*, IL-6, TNF-*α*, I*κ*B*α*, NF-*κ*B, p65↓, AMPK*α*, SIRT1↑	[116]
	Iran	In vivo	500 mg	NAFLD patient	ALT, hs-CRP, IL-6, NF-*κ*B, cytokeratin-18 M30↓	[117]
	Brazil	In vivo	30 mg/kg	HFD-induced mice	TC, TG, transaminases, insulin, TNF-*α*, IL-6, NF-*κ*B, ACC, PPAR*γ*, SREBP1↓	[118]
	China	In vivo	50 mg/kg	HFD-induced ULK1-deficient mice	IL-6, TNF-*α*, p65↓, I*κ*B*α*↑	[119]
Antifibrotic activity	China	In vivo	50 mg/kg	HFD-induced ULK1-deficient mice	Lipid droplets, the inflammatory infiltrate, ALT, AST, insulin, glucose, SREBP1c, MDA, 8-isoprostane↓, adiponectin, GPx↑	[119]
	Japan	In vivo	2, 20 mg/kg	HFD/LPS-induced mice	CD14, ALT, TNF-*α*, IL-6, p-STAT3↓	[120]
	Iran	In vivo	10 mg/kg	CCl_4_-induced rats	ALT, AST, ALP, hydroxyproline, LOX, TOS, MDA↓, TAC, –SH↑	[121]
Inducing autophagy activity	China	In vitro and in vivo	20, 40, 80 *μ*M; 0.4%	PA-induced HepG2 cells; HFD-induced mice	cAMP, SIRT1, pPRKA, P-AMPK, SIRT1↑	[122]
Regulating FXR activity	Iran	In vitro	25 mg/kg	HFD-induced rats	SIRT1, LXR, FXR↑, AST, ALT, ALP↓	[123]
